# A quantum–classical dual-track deep learning network for explainable Parkinson’s disease classification

**DOI:** 10.3389/frai.2026.1807209

**Published:** 2026-04-13

**Authors:** S. Alden Jenish, Arushi Pethkar, R. Karthik, K. Suganthi

**Affiliations:** 1 School of Electronics Engineering, Vellore Institute of Technology, Chennai, India; 2 Centre of Cyber-Physical Systems, Vellore Institute of Technology, Chennai, India

**Keywords:** convolutional neural network, deep learning, hybrid quantum computing, multi-modal image classification, Parkinson’s disease

## Abstract

**Introduction:**

Parkinson’s disease (PD) is a progressive neurodegenerative disorder caused by the loss of dopaminergic neurons in the substantia nigra, presenting with motor and non-motor symptoms in roughly 2–3% of the global population above age 60. Early detection is difficult because symptoms are subtle and often indistinguishable from normal aging. The MDS-UPDRS rating scale, the current clinical standard, is time-intensive, subjective, and requires experienced clinicians. Most computational approaches are unimodal and do not use image and structured clinical data in combination.

**Methods:**

We propose a hybrid quantum-classical dual-track multimodal network that classifies PD patients and healthy controls from hand-drawn spiral and meander patterns. The first track, the Topological Visual–Spatial Feature Encoder Network (TVSFE), uses a ghost module-based CNN with Cross-Dimensional Attention Bottleneck (CDAB) blocks incorporating coordinate attention, squeeze-and-excitation, and triplet attention, followed by a quantum variational circuit with amplitude embedding. The second track, the Variational Quantum Feature Mapping Network (VQFMN), encodes structured clinical and demographic data through RY rotation gates and strongly entangling layers. Outputs from both tracks are concatenated and passed through fully connected layers for classification.

**Results:**

On the HandPD test set, the model achieved 97.28% accuracy, 96.60% precision, 96.62% recall, and 96.54% F1-score, outperforming all CNN, transformer, and ML-based baselines compared. Five-fold cross-validation produced a mean accuracy of 96.58%. On the NewHandPD dataset, accuracy, precision, recall, and F1-score were all 95.45%.

**Discussion:**

The quantum-classical fusion outperforms both single-modality and fully classical variants. Grad-CAM localizes the spatial image regions driving classification and the perturbation-based sensitivity analysis identifies Root Mean Square (RMS) and age as the most influential structured features. Both together make the model’s reasoning traceable at the modality level, which is important for decision-making.

## 1 Introduction

PD is a neurodegenerative disorder that causes different motor and non-motor difficulties in the patient. It is a chronic and progressive disease and is estimated to affect nearly 2 to 3% of the global population around the age of 60. This creates a significant public health concern in the aging population of the world (Jankovic and Tan, 2020; Bernardo et al., 2021). The disease is characterized by the loss of dopaminergic neurons in the substantia nigra, causing low dopamine levels gradually. It also has a subtle nature during the early stages, causing low-level motor troubles such as tremor in the hand or stiffness of a limb. This affects the fine motor control needed to perform hand and arm movements. These early stages, when left unchecked, can lead to severe motor and cognitive function loss. Therefore, it is a challenge to clinically differentiate the early cues of PD from normal cognitive and motor changes that occur with aging (Srinivasan et al., 2024).

The clinical diagnosis of PD is conventionally determined by neurologists using applied standards such as Movement Disorder Society (MDS) diagnostic criteria. The progression of the disease is measured using the MDS Unified Parkinson’s Disease Rating Scale (UPDRS) (Chandra et al., 2021). Clinical features can be subtle or nonspecific, or they can be indistinguishable from other neurodegenerative conditions, especially during the early or prodromal phases. The system of identifying PD takes significant time due to a lack of clear biological markers, or the progression, or resolution, of other features will be observed before classic motor symptoms are observed. Currently, Computer-aided Diagnosis (CAD) systems are utilized to address the issues with traditional diagnostic approaches for PD (SasiRekha et al., 2025). ML and Deep Learning (DL) techniques can help find complex patterns in clinical data, offering more consistent support for diagnosis when used along with traditional assessments by clinicians (Anisha et al., 2023). DL architectures are particularly effective at learning hierarchical representations from raw data with minimal manual processing and thus have been shown to improve the sensitivity of models for PD detection. Although ML and DL methods were shown to have significant potential for automated analysis and early detection of PD, they have limitations that prevent them from being fully implemented in the clinical space. In scenarios that consist of small datasets or data with unbalanced distributions, it leads to overfitting of the model. Such models present biased decisions, making them unable to learn important and subtle features of early-stage PD. Additionally, large DL architectures are often heavy in computations and bring computational limitations to deploying them in real-time diagnostic settings where there is resource limitation (Patil and Ford, 2024; Tabashum et al., 2024).

To mitigate the limitations, current research explores alternative computing strategies to yield significant improvements over existing works. From which, quantum computing has been explored as a potential alternative by leveraging its high-dimensional and complex data pattern recognitions. Quantum computing modifies data based on state principles that are different from classical systems, namely, quantum superposition and entanglement. Within the domain, Quantum Machine Learning (QML) has gained interest in helping models to leverage quantum advantages in learning tasks. QML models can learn deep, intricate details and decision boundaries for improved performance results with a limited amount of data. Similarly, quantum-inspired algorithms, which approximate quantum performance strategies to improve learning efficiencies while also assisting in computing, have also presented promising results. In this study quantum computing is used to classify PD from both image and numerical samples. The model architecture is trained on data from hand-drawn patterns collected from both individuals with PD and healthy individuals, allowing the model to learn patterns of symptoms of motor impairment related to the disease.

## 2 Related works

Researchers have explored various data modalities to develop systems for the classification and diagnosis of PD. These modalities include speech recordings, gait analysis, handwriting patterns, and biomedical signals such as Electroencephalogram (EEG), Electromyography (EMG), and neuroimaging data. Each data modality will capture different biomarker data related to PD and allow researchers to develop diagnostic models for the specific data modalities. For instance, speech-based techniques account for vocal tremors and aberrations in articulation, and handwriting studies measure motor control and micrographia. Gait and mobility data have commonly used wearables or video data to account for the motor symptoms of PD. Some recent studies have used multimodal methods in research protocols that apply different data modalities to improve aspects of diagnostics. This section reviews existing contributions that were presented for the classification of PD.

Among various modalities explored in literature, several studies have incorporated neuroimaging data for the classification of PD. Camacho et al. (2024) presented a simple fully Convolutional Neural Network (CNN) for PD diagnosis. The study utilized 3D Diffusion Tensor Imaging (DTI) maps, 3D T1-weighted scans, 3D log-Jacobian maps, and demographic features such as age and sex for training the system. The study also highlighted important regions using heatmaps generated using saliency maps for explainability. Yang et al. (2021) also employed similar multi-modality data from T1-weighted and DTI images to classify PD from healthy subjects. The study utilized an ensemble model stacked with Support Vector Machine (SVM), Random Forest (RF), K-Nearest Neighbors (KNN), Artificial Neural Network (ANN), and Linear Regression (LR). Chan et al. (2022) also used DTI input data for early detection of PD. The research employed a graph neural network featuring an attention mechanism to integrate multi-modal imaging data and multi-omics groups to predict PD. Ma et al. (2021) proposed a Recurrent Neural Network (RNN) with attention for detecting PD. The research used five brain area features from MRI and DTI alongside clinical and exercise data for training and examining the analysis of the classification. Chen et al. (2023) formed an ML model based on extracted DTI data with patient clinical demographics to predict PD. The research involved a feature selection step from the extracted DTI data using RF and Spearman’s rank correlation analysis. Prasuhn et al. (2020) also included an ML-based tradition with DTI data for PD detection. Standard diffusion-based metrics derived from DTI are based on 3D diffusion as a function of spatial location processed from the DTI imaging. Hussain et al. (2023) introduced a deep CNN with neuroimaging data for PD classification. The authors presented a method to address the problem of overfitting with the limited dataset in their analysis.

Hathaliya et al. (2022) suggested a 2D CNN for multiclass classification of PD. The model presented in the research was computationally less expensive, yielding a substantial improvement in performance, like most pre-trained models, under the same constraint. Further, Hosseinzadeh et al. (2023) used ML techniques to build predictive models for the Montreal Cognitive Assessment (MoCA) score at 4 years following diagnosis (year 4) by leveraging both clinical and imaging data. The study investigated different classifiers and used feature selection, both of which increased prediction accuracy by detecting meaningful structure while minimizing the effects of overfitting. Similarly, Adams et al. (2021) suggested a CNN technique to predict a patient’s year-4 motor function score using only Dopamine Transporter Single Photon Emission Computed Tomography (DAT SPECT) images and non-imaging clinical measures. This method improved estimates of motor function in PD through EEG features while skipping image segmentation and feature extraction, greatly increasing ease of application. Hsu et al. (2020) presented the performance analysis of state-of-the-art models such as AlexNet, GoogLeNet, VGG, DenseNet, and a residual neural network. Two sets of SPECT image datasets, grayscale and pseudo-color, were used to train and test the models. Castillo-Barnes et al. (2020) implemented morphological feature extraction from I-Ioflupane SPECT scans for PD diagnosis. Feature selection algorithms with SVM were utilized for efficient classification and reliability of the biomarkers extracted from the scans. Similarly, Acton and Newberg (2006) also incorporated SPECT scans for PD classification. Feature extraction was employed to extract the striatum and striatal pixel values from the input scans, and the extracted features were used to train a three-layer ANN model.

Structural MRI, particularly T1- and T2-weighted sequences, has been employed to classify PD based on structural changes accompanying its development. Akram and Kumar (2024) recently developed a unique DL architecture to alleviate issues such as complexity, noise samples, and redundant features. The image scans underwent preprocessing through wavelet transforms, and important features were selected with the help of an optimization algorithm. Majhi et al. (2024) proposed an enhancement in the classification process using a metaheuristic optimization algorithm. The study provided an analysis of the performance of various DL models that were enhanced through the Grey Wolf Optimization (GWO) technique.

Bu et al. (2023) employed T1-weighted imaging and Susceptibility Weighted Imaging (SWI) to identify idiopathic Parkinson’s disease and Multiple System Atrophy (MSA). The study extracted features using PyRadiomics, and the features were reduced via LASSO. Furthermore, the Shapley Additive Explanations (SHAP) analysis was used to identify which features were important, once again based on features extracted in the prediction. Gore et al. (2023) outlined an ML model trained using radiomic features obtained from four different Local Binary Patterns (LBP). The Recursive Feature Elimination (RFE) for feature selection was also implemented, and summaries of the performance of SVM and RF, using the selected features, were presented. Betrouni et al. (2021) used T1-weighted MRI images and clinical scores for diagnosis of PD. The study extracted first- and second-order texture-based features from the MRI images and used LASSO regression for feature selection. Shimozono et al. (2024) used radiomic features obtained from T1-w/T2-w ratio images. These ratio images were created using MRTool and the statistical parametric mapping toolbox. Additionally, the study performed a feature selection method using the LASSO regression model and a heatmap visualization to identify important radiomic features. Panahi and Hosseini (2024) also carried out a similar analysis in PD diagnosis using radiomic features extracted from multi-modality images of 16 brain regions of interest. The radiomic features were obtained from the conjunction of DTI and T1-weighted imaging. The study also performed a three-class and binary classification study of PD using the Light Gradient Boosting Machine (LGBM) classifier. Tassew et al. (2023) performed ROI detection using an ensemble of U-NET and You Only Look Once (YOLO) algorithms to detect ROIs from DaTScan and T2-weighted MRI images. The study implemented mosaic augmentations to add different imaging variants and context that helped improve the generalization ability. Cui et al. (2023) proposed a DL architecture that utilizes an adaptive weighted attention mechanism to detect PD efficiently. Additionally, the model was equipped with multi-branch feature processing modules utilizing MRI T2 image slices for diagnosing PD.

Alniemi and Mahmood (2023) employed a custom CNN-based architecture for PD classification from healthy subjects using hand-drawn spiral images. Parisi et al. (2021) implemented a CNN-based architecture that utilized a hyper-sinh activation function. The hyper-sinh activation helped improve automated pattern finding and discrimination capabilities of the model. The study also presented the performance analysis of different activation functions. Li et al. implemented a custom CNN classification network based on a continuous convolution mechanism (Zhu et al., 2022). The study also incorporated a time-series classification of PD and highlighted the important features identified by the model.

Shanthini and Chandrasekar (2025) proposed the multi-modal PD classification using hand-drawn images and voice signals. Various features were extracted from the preprocessed signals and augmented images and were reduced to a subset of useful features using the Ladybug Hawk optimization algorithm. Das et al. (2022) employed a single-level 2-D discrete wavelet transform and feature extraction for detecting PD. The histogram of oriented gradients and coefficients was extracted from the transformed images, and the performance analysis of various ML models was presented. Pereira et al. (2016a) utilized image preprocessing techniques for the extraction of a suitable representation of hand-drawings of patients from the template. The study also presented performance metrics of various ML models under different simulated experiments to find the efficient utilization of the extracted features. Afonso et al. (2019) employed performance analysis of models such as Optimum-Path Forest (OPF), CIFAR10 net, LeNet, and ImageNet models based on patterns learned from the output of a CNN or raw images.

Özdemir and Özyurt (2025) proposed a ViT transformer model coupled with an ElasticNet future selection algorithm. ViT transformers were used to extract the features from the drawing images, and training was carried out using various ML models. The SHAP analysis was also incorporated, highlighting the impact of DL-derived features extracted. Wang et al. (2023) presented a Swin Transformer model in addition to a coordinate attention module for PD classification. The study utilized CycleGAN for the augmentation of input image samples and a transformer to extract fuzzy edge information. Dogan (2024) presented the performance analysis of nine different transfer learning models for the diagnosis of PD. These transfer learning models were used to extract the features of the input images, and Neighborhood Component Analysis was employed to select the best features. The selected features were trained using SVM for the final classification process. Wang et al. (2024) implemented an LSTM-CNN architecture to classify hand drawings from PD patients and healthy controls. The study utilized two sets of datasets and a sequence segmentation technique to preserve the local temporal features of the images.

Bahaddad et al. (2022) discussed a combination of a DL-based classification with a metaheuristic strategy. The research developed an advanced version of the Sailfish Optimization (SFO) algorithm for feature selection and applied the Bidirectional Gated Recurrent Unit (BiGRU) for the final classification task. Mansour (2024) also used a metaheuristic feature selection algorithm applied to create a successful feature subset for PD classification. The study employed a novel method using quantum computing to apply the Mayfly Optimization Algorithm (MOA), integrated with a new hybrid CNN architecture, to provide the final answer. The present study has identified significant weaknesses in the current literature, such as lack of multimodality incorporation, only using handcrafted representation of features, and using traditional architectures that were never developed as architectures to accommodate the nature of handwriting data. The following section summarizes these limitations and describes the strategies used to alleviate these limitations in this study.

### 2.1 Research gaps and motivation

The following research gaps are addressed in the proposed work:

1. Many existing studies incorporate predefined or custom feature selection techniques that may not entirely encapsulate the complex variations within the hand-drawn data. Allowing the model to work on a raw and complete representation of data may provide more insight on the complexity and the disabling motor impairments of people affected by PD.
2. Existing studies offer limited exploration of multimodal inputs in combination with hand-drawn images, leaving the potential benefits of integrating complementary data sources underutilized. There is a lack of exploration into layered or attention-based fusion frameworks that combine image features with contextual patterns hierarchically or adaptively.
3. Most of the existing studies on PD detection predominantly leverage either standard CNN architectures or pre-trained models. Designing a custom architecture that is tailored to the unique aspects of data has the potential to improve the model’s generalization ability substantially.

### 2.2 Research contributions

The proposed study presents novel contributions to address the identified gaps in existing research. The contributions presented are:

1. The research presents a multimodal architecture that utilizes hand-drawn diagnostic images and structured data. The architecture consists of two tracks, namely, the TVSFE track and the VQFMN track, which leverage visual patterns and patient clinical data, respectively. This helps the model achieve a comprehensive understanding of the patient data and helps improve the detection performance between PD and healthy individuals.
2. The VQFMN track directly encodes raw structured data into quantum stages. This enables the network to leverage quantum principles to capture complex and unique patterns encoded in the data without the need for intermediate feature engineering. The model can learn important patterns from the data by utilizing the representational power of the quantum circuit.
3. The TVSFE track leverages attention-based CNN architecture coupled with a quantum variational circuit for efficient feature extraction and processing. The track consists of Cross-Dimensional Attention Bottleneck (CDAB) blocks that utilize Coordinate Attention (CA) and Triplet Attention (TA) mechanisms for discrimination of subtle and unique spatial features.

## 3 Proposed system

**Figure 1:**
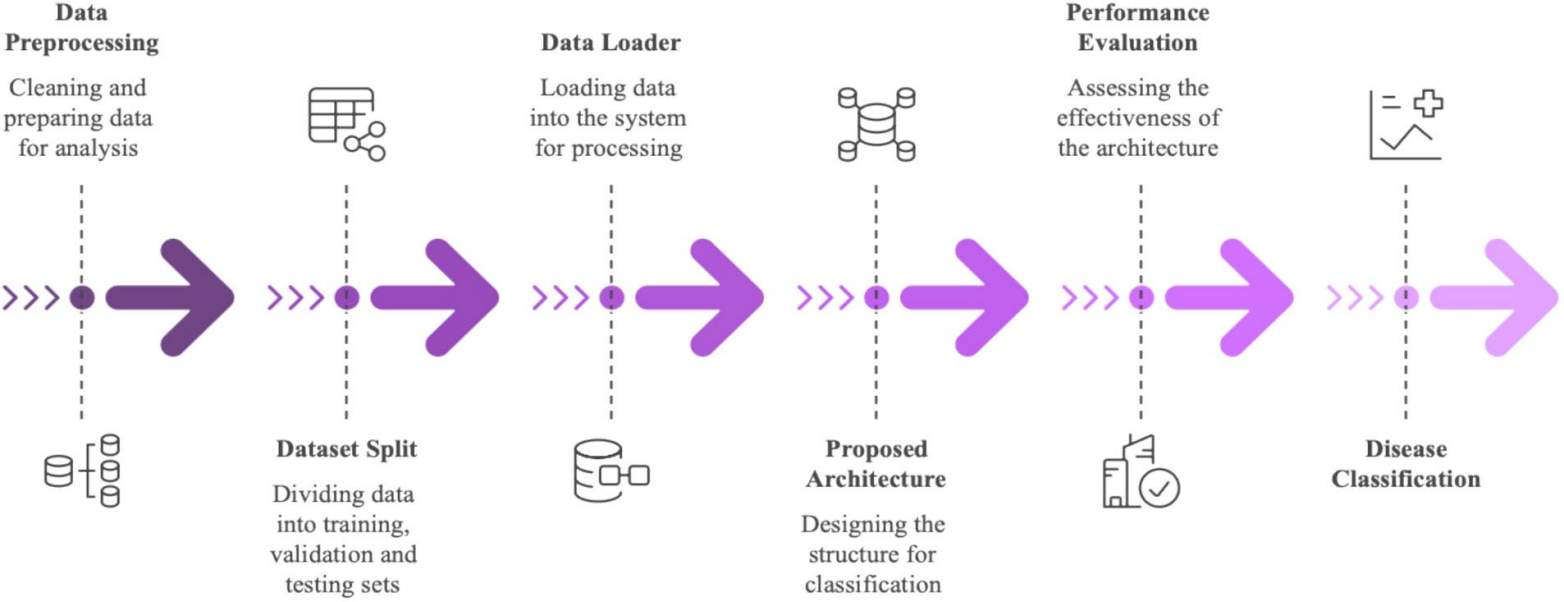
Workflow of the proposed approach.

This section presents the overview of the proposed workflow, highlighting the stages from dataset preprocessing to model assessment, as illustrated in Figure 1.

### 3.1 Dataset description

**Figure 2:**
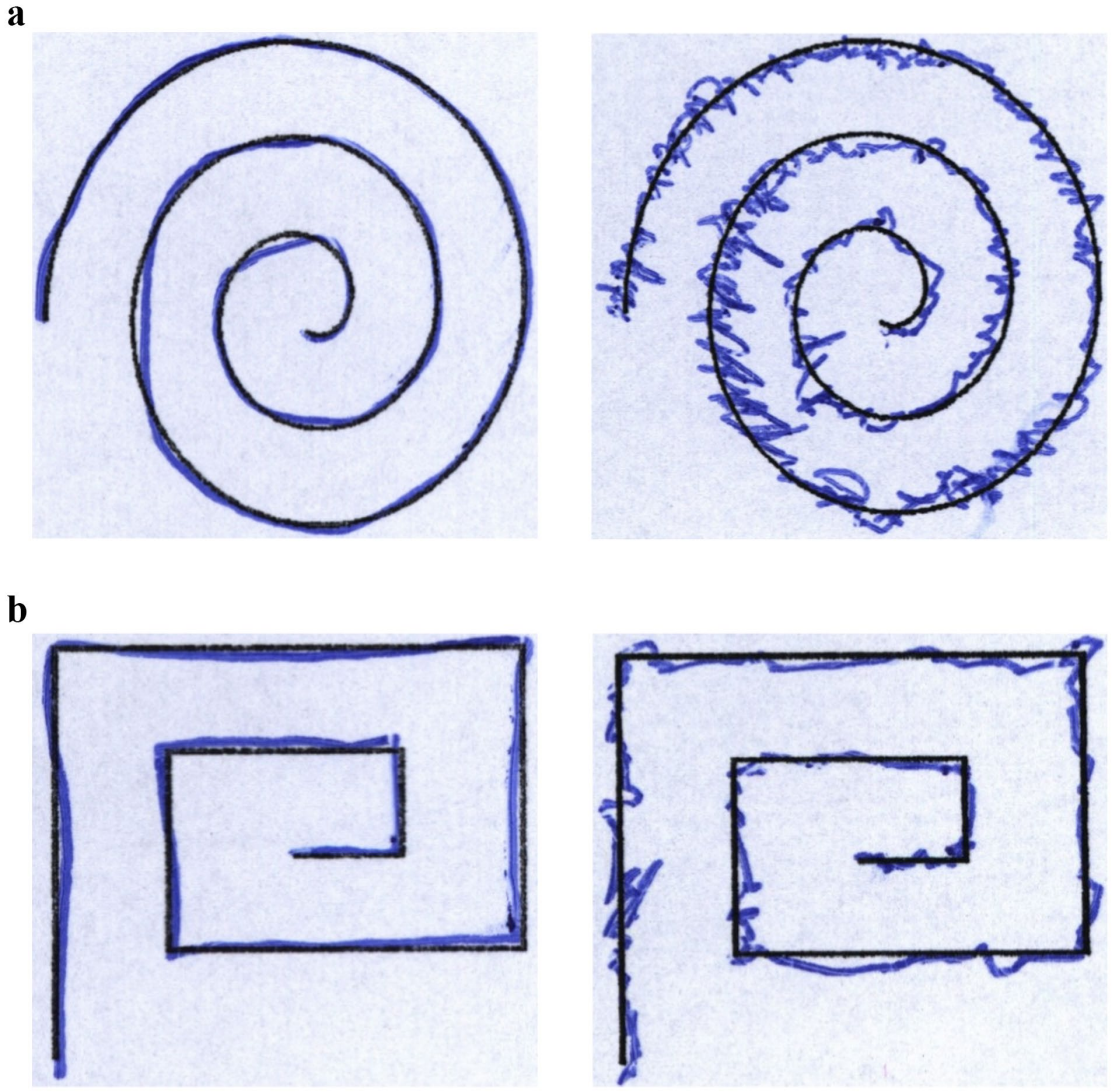
**(a)** Sample of spiral drawings of the control subject and the PD patient, respectively. **(b)** Sample of meander drawings of the control subject and the PD patient, respectively.

The HandPD is a publicly accessible dataset that includes handwriting evaluation data for diagnosing PD. The dataset was developed by the Botucatu Medical School of São Paulo State University in Brazil. From 92 subjects, handwritten contributions were made from 18 healthy controls and 74 PD patients. Each participant was asked to perform tasks where they drew four spirals and four meanders, resulting in a total of 736 images, 368 of each spirals and meanders. The healthy subjects consist of 6 male and 12 female subjects aged 19–79. Similarly, the PD group consists of 59 male and 15 female subjects aged 38–78. The first version of the dataset consists of images collected from a total of 35 examinations, with spiral and meander drawings (Pereira et al., 2016a). These drawings are divided into two groups, which contain PD patients and healthy controls. The dataset also has collected CSV files that contain specific metadata for each particular sample. These files contain attributes like exam ID, image name, patient ID, class label, gender, handedness, age, and handwriting-derived features such as Root Mean Square (RMS), Mean Relative Tremor (MRT), and the standard deviation of the differences between the exam template and the handwritten trace. These files make it easy to use the data in statistical analyses and machine learning applications without needing extra preprocessing. The second and newer version (NewHandPD) was also released, comprising all samples of the first version, along with their respective signals in their raw format (Pereira et al., 2016b). These signals were collected from the sensors attached to each individual’s pens during the examination. Table 1 presents the information about both the datasets. Samples of the drawings are illustrated in Figures 2a,b.

**Table 1:** Summary of the dataset related details.

Dataset	No. of samples		No. of participants		Average age (years)	
	Healthy control	PD patients	Male	Female	Healthy control	PD patients
HandPD	144	592	60	27	44.22 ± 16.53	58.75 ± 7.51
NewHandPD	280	248	39	27	44.05 ± 14.88	57.83 ± 7.85

### 3.2 Data preprocessing

**Figure 3:**
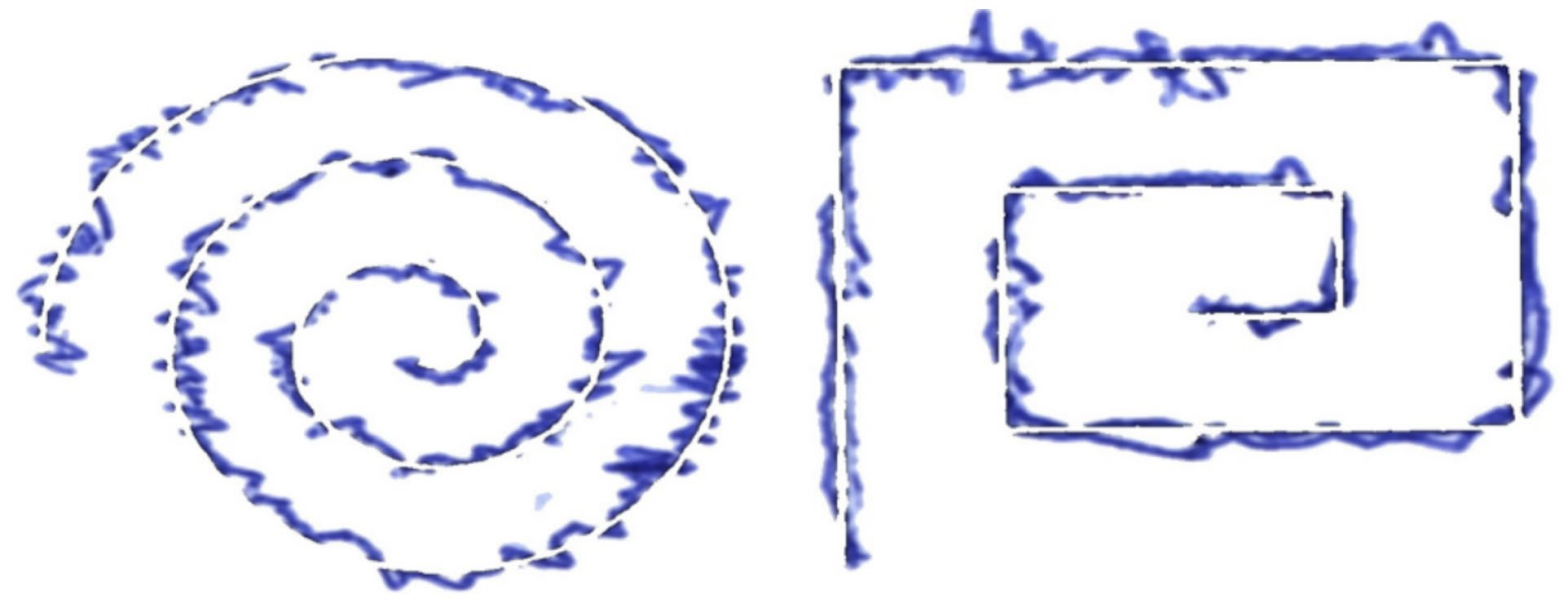
Illustration of pre-processed image samples.

This section discusses the preprocessing methods that were incorporated before forwarding the dual modality, comprising hand-drawn images and their associated structured input data, to the proposed system. The visual modality inputs undergo a multi-step preprocessing pipeline to enhance contrast and suppress background noise. Mean and median filters were used to balance the local noise reduction while preserving structural edges important for classification. A grayscale suppression algorithm is applied to isolate the patient’s hand-drawn trace from the exam template. The pixels are considered for suppression if the inter-channel differences across red, green, and blue channels fall below a perceptual threshold and are given by Equation 1.


$$\text{grayscale}(i)=\{\begin{array}{l} 1\;\text{if}\;|R(i)-G(i)|<45\;\text{and}\;|R(i)-B(i)|<45\;\text{and}\;\\ \;\;\;\;\;\;\;\;\;\;\;\;\;\;\;\;\;\;\;|G(i)-B(i)|<45\\ 0\;\text{otherwise} \end{array}$$ (1)

where R(i),B(i), and G(i) represent the red, blue, and green channel intensity of pixel i respectively. The operation ∣⋅∣ denotes the absolute value operator. The threshold value is selected to ensure the hand-drawn trace is separated from the rest of the exam template. Converting pixels that represent the trace into white pixels reduces the contribution from the non-informative background and maximizes the contribution from the relevant structural features necessary for the model to make predictions. Next, the images are processed back up, or restored, and resized to maintain size uniformity in the study mapping images to a dimension of 512×512 pixel-sized images. Figure 3 depicts sampled images of preprocessed spiral and meander images. The structured metadata file accompanying each sample provides clinical information of the subject and statistical measurements derived from the imaging in the process. The features were normalized in the range [0,π] and rescaled to the interval to accommodate the input requirement of the quantum circuit. This mapping aims to preserve the relative importance of each feature while accommodating compatibility behind quantum rotation-based encodings.

### 3.3 Proposed network

**Figure 4:**
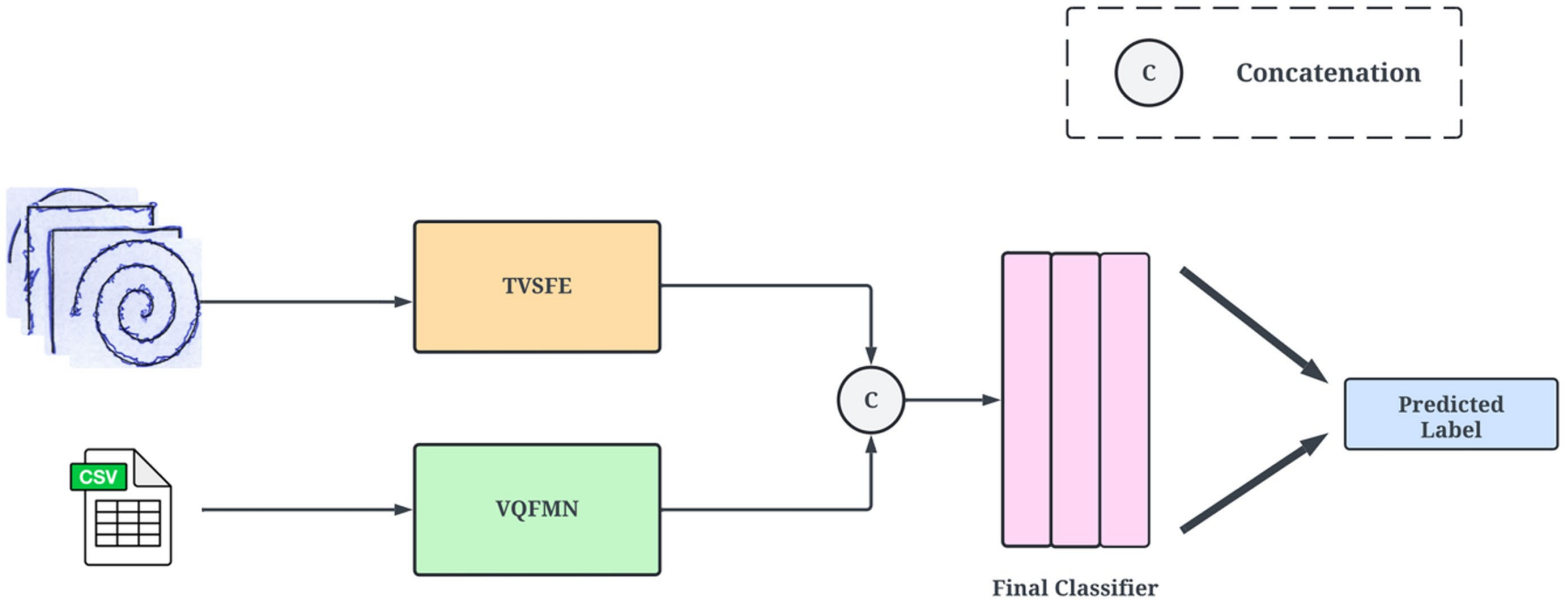
Overview of the proposed architecture.

The proposed architecture utilizes a two-track multimodal network for the classification of PD. The first track comprises the TVSFE. The second track utilizes the quantitative data derived from the dataset and consists of the VQFMN model. Figure 4 presents the overview of the architecture. TVSFE leverages visual patterns such as tremors and irregularities from spiral and meander drawings to distinguish control and PD subjects. Through the incorporation of various attention mechanisms and a quantum circuit, the track extracts hierarchical features from low-level edges to high-level specific to disease characteristics. The network learns subtle visual cues such as line thickness irregularities and motor impairment indicators of PD by inferring information from these spatial visual observations. Utilizing the classical CNN component with the quantum-enhanced component enhances the model’s discriminating ability to make accurate predictions on complex heterogeneous image data.

The quantitative characteristics are pre-processed using a simple imputer and normalized to a feature range of [0, π ]. This normalized range is compatible with the quantum circuit and aligns with the angular inputs often used with quantum embeddings. The quantum neural network will have a high-dimensional quantum feature space that extracts the complex features. The enhanced ability of the model to learn complex numerical patterns is due to the series of entangling gates in the module. The series of entangling gates allows for an increase in classification performance when accessing and encoding heterogeneous components of data into class labels according to the complex characteristics.

### 3.4 Topological visual–spatial feature encoder network

The TVSFE employs visual attributes from drawing samples to distinguish between control and PD subjects. The processing through the network starts with a convolutional layer of 7×7, which is specialized to extract low-level visual features from the input images. The relatively large size of the kernel is capable of capturing broader spatial information and establishes a solid foundation for further hierarchical feature extraction. It is then followed by a batch normalization and ReLU activation function to achieve a stabilized gradient flow process.

Subsequently, the network progresses into a series of MAGE blocks. These blocks utilize Ghost Module (GM) to generate additional feature maps while maintaining low computational operations. It is followed by attention mechanisms that help capture subtle cues important for PD detection. The initial set of CDAB blocks leverage CA and squeeze-and-excite mechanisms to learn spatial locations and channel importance. The later blocks utilize the TA mechanism to capture efficient feature maps along the height, width, and channel axes. This enables the model to improve discriminative capabilities and spatial relevance. In addition, residual connections were integrated to preserve feature integrity and smooth gradient flow through deep connection layers.

**Figure 5:**
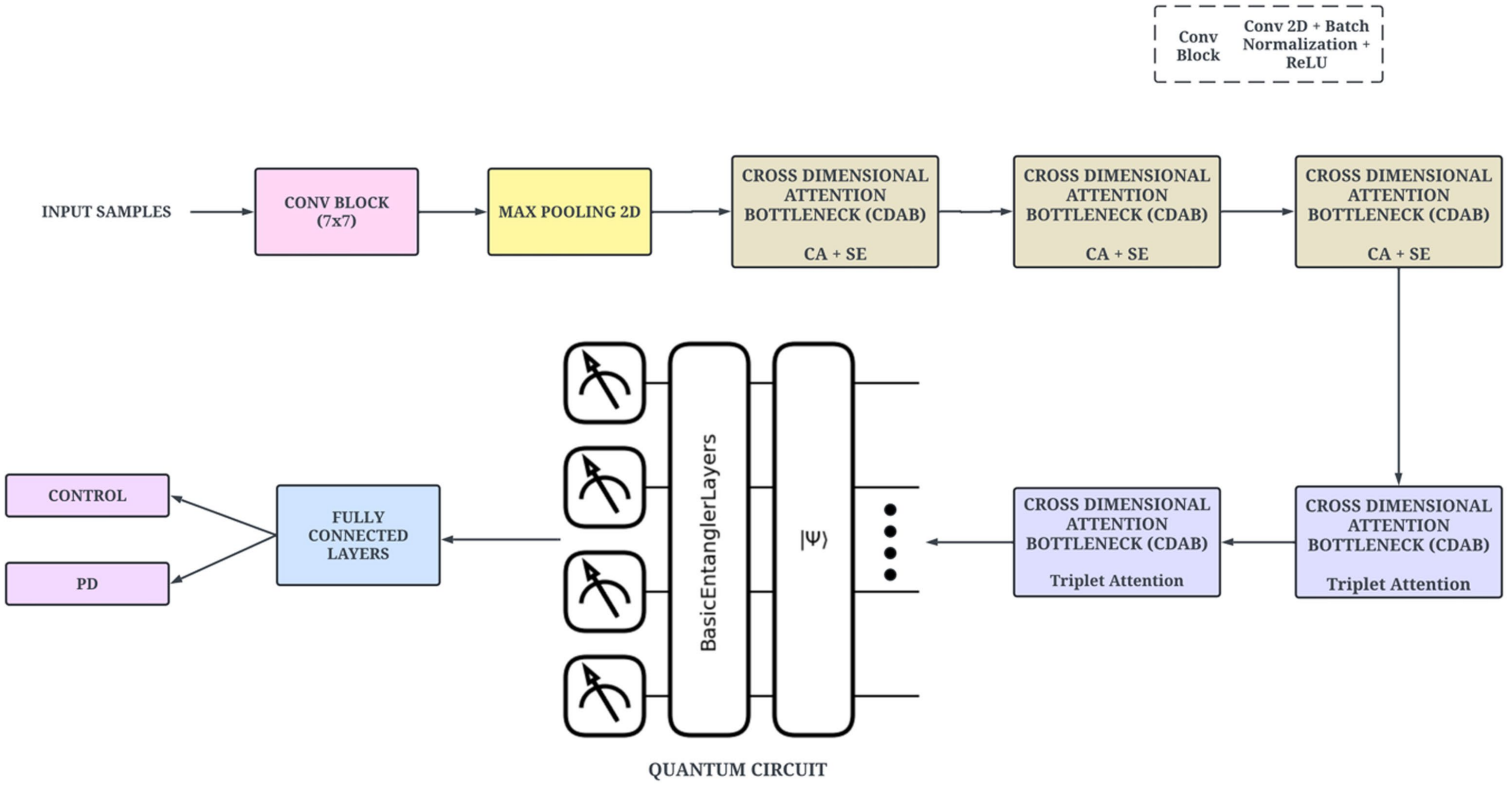
Schematic of proposed TVSFE network.

After the convolutional parts of the network, Global Average Pooling (GAP) is used to reduce the spatial dimension into a smaller feature vector, focused on the most informative aspects of the image. The resulting feature vector is then passed through interconnected layers with LeakyReLU activation before it outputs a 256-dimensional embedding of the image. Layer normalization and L2 normalization are then passed onto the generated embedding of the image to keep the components in the same scale and to improve generalization. The normalized 256-dimensional feature vector is passed into a quantum variational layer implemented using PennyLane. This layer employed Amplitude Embedding to encode this feature vector into a quantum state of 8 qubits. Subsequently, a series of entangling layers are implemented via parameterized gates. The Pauli-Z measurements are utilized to derive the output vector that consists of quantum expectation values. Figure 5 illustrates the proposed structural design of the network.

#### 3.4.1 Cross-dimensional attention bottleneck (CDAB)

**Figure 6:**
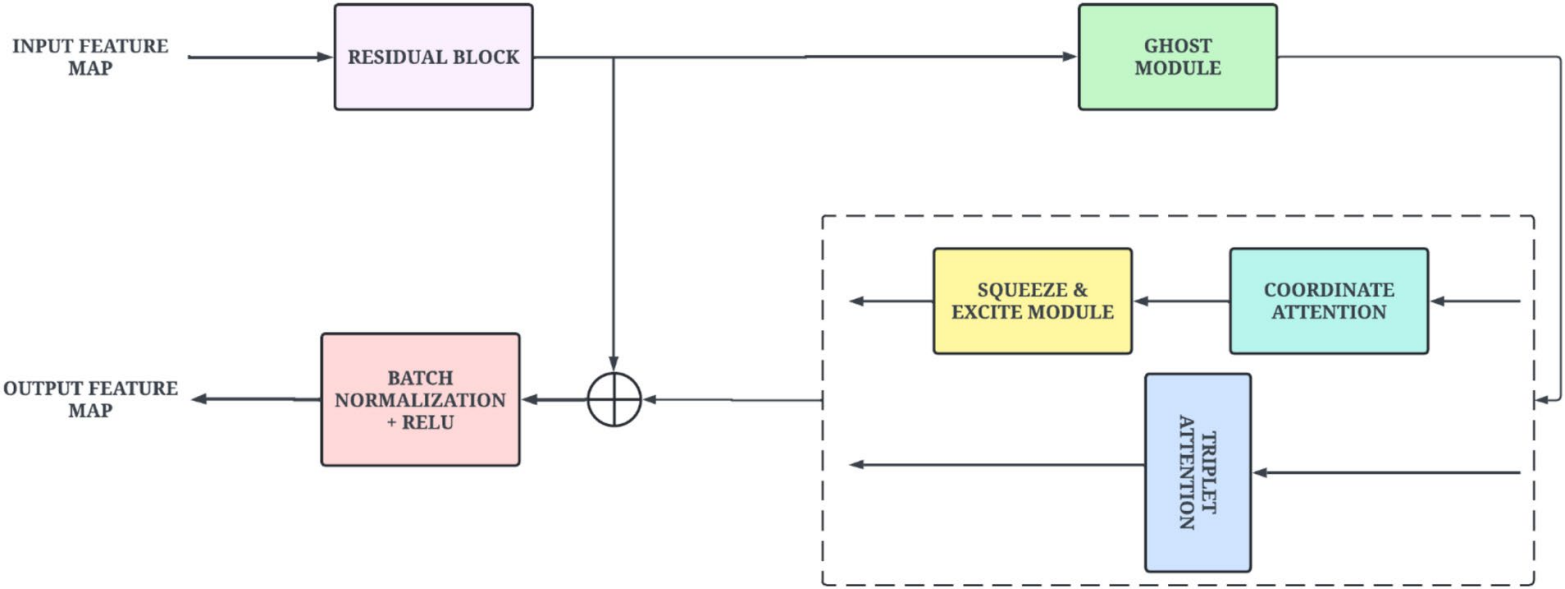
Proposed architecture of CDAB block.

The CDAB block is a key building block of the TVSFE track exhibiting efficient and attention-enhanced convolutional purposes. It utilizes GM, which is a lightweight convolution-based approach, to help minimize computational redundancies from calculating the feature maps by combining several standard convolutions and inexpensive linear operations. It utilizes dynamic characteristics of attention to support efficient and optimal feature representations. In the upper level of the definition, the CA and SE adaptation mechanisms are employed sequentially. The purpose of the CA module is to allow the model to systematically focus on the spatial locations of significance by encoding the positional information in the channel attention. The SE module recalibrates channel-wise responses to signify informative channels over less informative ones. The block also seamlessly integrates a triplet attention mechanism alternatively to capture interactions along three orthogonal planes that enable rich multi-dimensional attention modeling in parallel with a minimum of parameter overhead. Moreover, the CDAB block employs a residual connection that permits input features to bypass the primary convolution-attention path. This preserves integrity between low-level features and efficiently facilitates gradient flow where the output and input dimensions differ via strides and channel expansions. The outputs of the divergent paths are summed and normalized, making the learning process stable and fastening convergence. Figure 6 presents the structural design of the CDAB block.

### 3.5 Variational quantum feature mapping network

**Figure 7:**
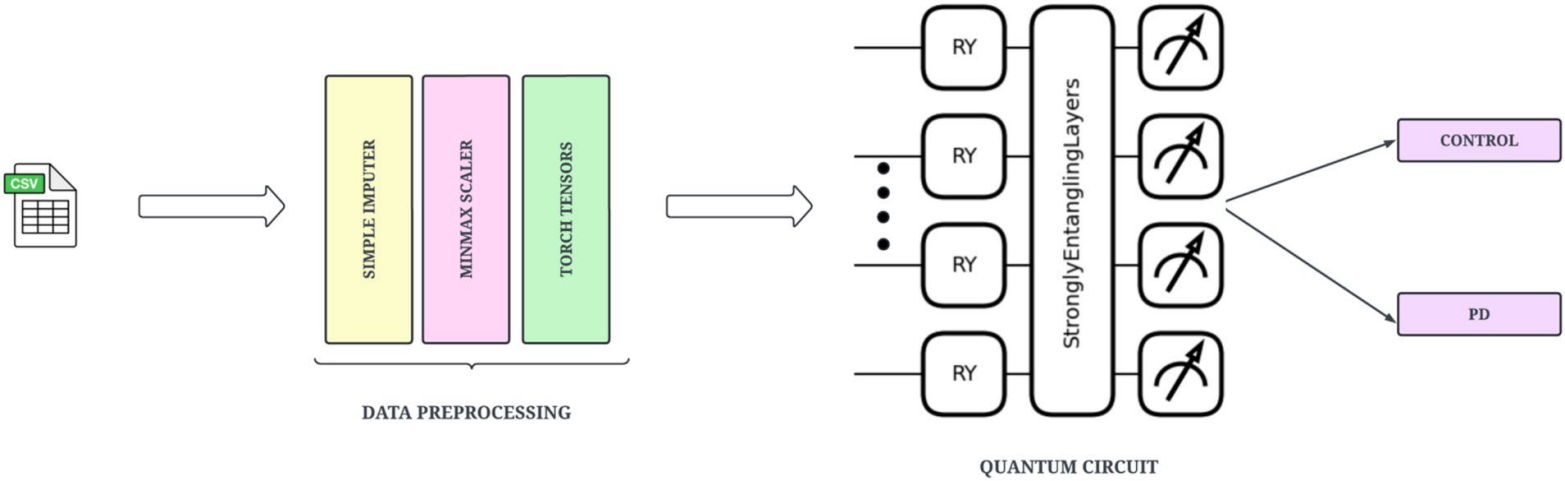
Illustration of VQFMN.

The VQFMN represents a second computational track of the proposed workflow. The VQFMN is designed to take advantage of quantum computing to capture complex non-linear interactions in structured data. This circuit can encode each input feature into quantum state space with the application of RY rotation gates. The method will be effective at preserving feature-wise significance across multiple samples. The network then applies strongly entangling layers to produce entanglement of all qubits. This layer is responsible for introducing parameterized rotation gates and entangling operations, allowing the network to learn high-dimensional feature interactions. The outputs are extracted as a vector of expectation values of Pauli-Z operators. The architecture of the proposed VQFMN is shown in Figure 7.

### 3.6 Classification

The feature representations from the TVSFE and VQFMN tracks are concatenated to create a complementary single embedding of the multimodal data. The TVSFE captures spatial and texture-based features from image samples, while the VQFMN utilizes high-dimensional quantum feature space for finding complex nonlinear relationships. The output from these tracks is normalized to ensure smooth compatibility for the unified feature space. The final representations are passed to fully connected dense layers to model over visual and structured inputs. Results are presented through the softmax layer, which provides whether the input patient is affected by PD or is a healthy individual.

## 4 Experiments and results

This section presents the details about the environmental setup and experimental configurations that were used while training the proposed system. It also presents a series of experiments and their results to find the specific impact of each module used in the proposed network.

### 4.1 Environmental setup

The proposed system was implemented using PyTorch and PennyLane frameworks. All the experiments were trained and validated on Kaggle’s cloud-based environment. The focal loss function was utilized to address the class imbalance, and the ReduceLROnPlateau learning rate scheduler was employed for allowing model to attain a smooth convergence. In addition, early stopping was incorporated to help avoid overfitting to the training data and stability throughout the process.

### 4.2 Hyperparameter tuning

DL models require suitable hyperparameter tuning to improve the model’s ability to provide efficient classification performance. Table 2 presents the summary of the hyperparameters considered in the research and their optimal values obtained during testing. Optimization was carried out using the Adaptive Moment Estimation (Adam) optimizer with an initial learning rate between 1e-3 to 1e-5 due to its adaptability and effectiveness for optimizations where the gradients are sparse. A learning rate scheduler was also employed to minimize oscillations and improve convergence. An early stopping mechanism was also employed to avoid overfitting, ensuring termination of training once the validation performance plateaued.

**Table 2:** Summary of the hyperparameter tuning process.

Hyperparameters	Values/Methods tested	Optimal parameters
Batch size	Chandra et al. (2021); Tabashum et al. (2024); Hathaliya et al. (2022); Parisi et al. (2021)	4
Optimizer	Adam, RMSProp, SGD	Adam
Learning rate	[1e-3, 1e-4, 5e-4, 1e-5]	1e-4
Learning rate scheduler	ReduceLROnPlateau, CyclicLR	ReduceLROnPlateau
Loss function	Focal Loss ( γ=2) , Focal Loss ( γ=3) , Cross-Entropy Loss	Focal Loss ( γ=3)

### 4.3 Ablation studies

This section presents the details of the ablation experiments conducted in the study to evaluate the efficiency and contribution of different modules included in the proposed architecture. This individual assessment of the components helps researchers understand the impact of each design presented in the network, helping with future extensions of the work.

#### 4.3.1 Analysis of the topological visual–spatial feature encoder network (TVSFE)

**Figure 8:**
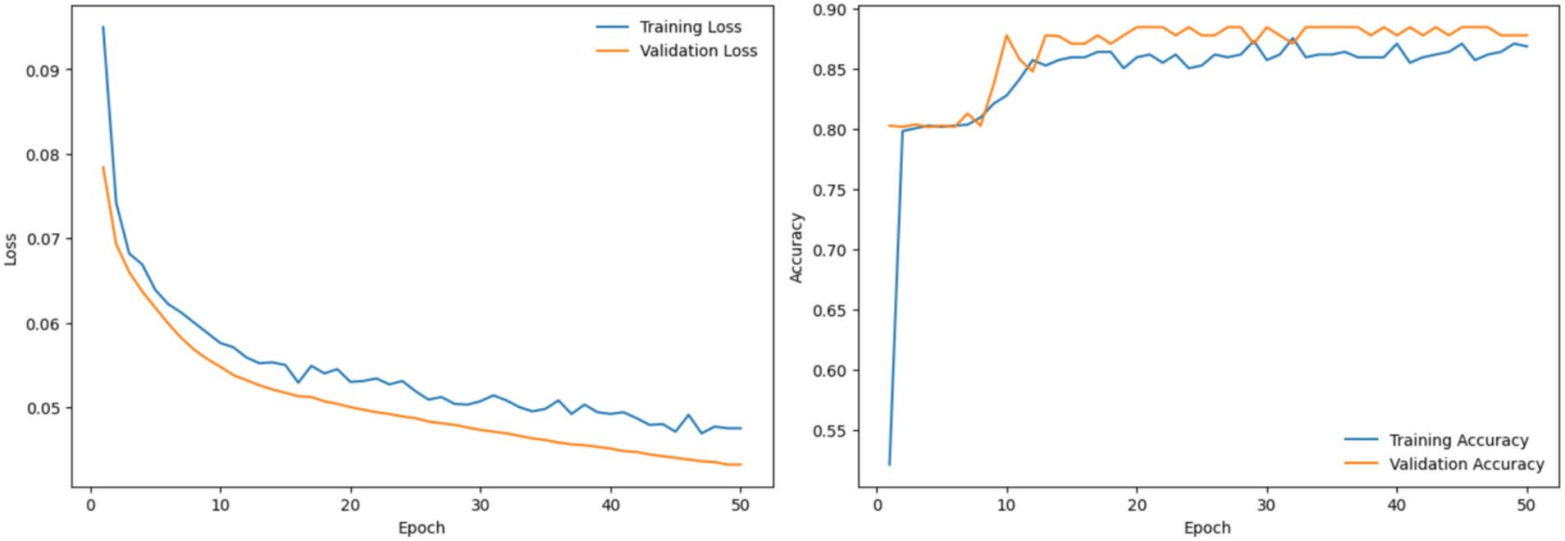
Analysis of TVSFE using loss and accuracy graphs.

This section presents the performance evaluation of the TVSFE track in the proposed architecture. The TVSFE track utilizes an attention-enhanced CNN with a variational quantum circuit to analyze hand-drawn diagnostic images of patients. The circuit allows the model to further optimize the feature maps that were obtained from the CNN modules in order to capture complex and nonlinear patterns in the quantum feature space. The network attained an accuracy score of 93.93% when tested with unseen samples, which provided some indication of the model’s ability to accurately identify subtle features for detection of both PD and healthy patients. The plots observed for the epoch-wise loss and accuracy are shown in Figure 8.

#### 4.3.2 Analysis of the variational quantum feature mapping network (VQFMN)

**Figure 9:**
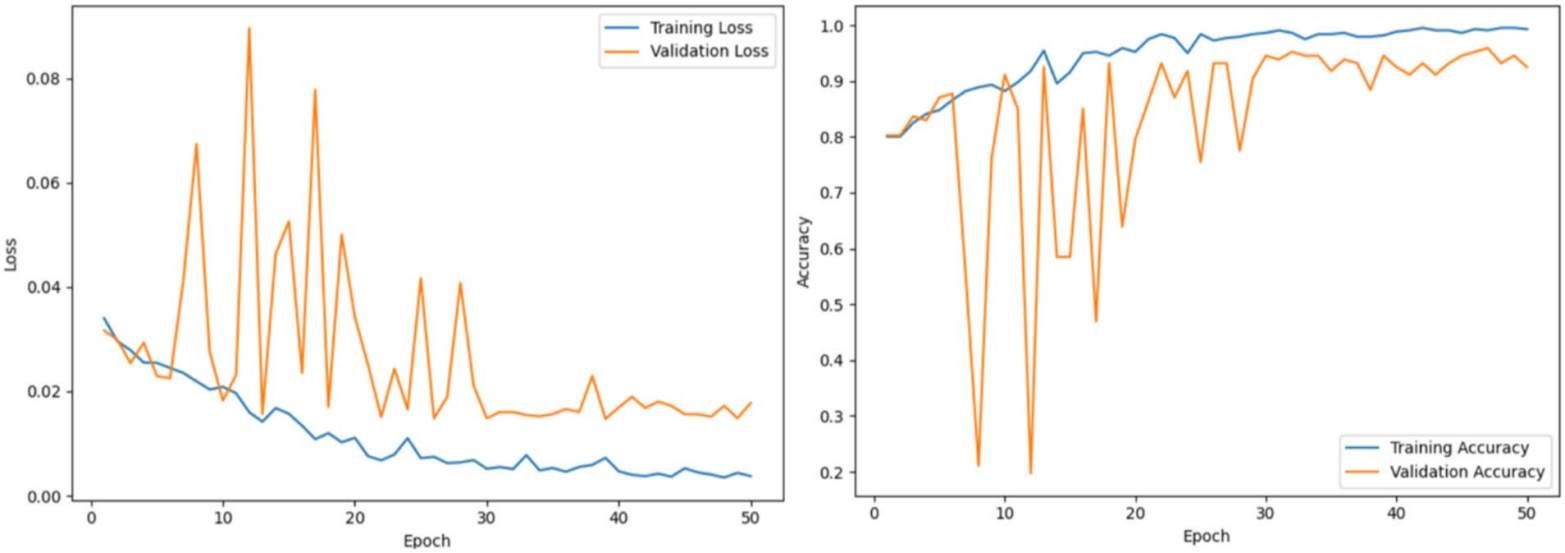
Loss and accuracy graphs of training and validation sets.

This experiment presents the performance obtained from using only the quantitative modality data of patients and healthy subjects. The VQFMN employs angle embedding and strongly entangling layers that transform numerical input into a high-dimensional quantum space. The strongly entangling layers are a variational quantum architecture that captures higher-order correlations and non-linear interactions within the feature space. This facilitates richer representations of structured data that may be suboptimal for classical encoders. The accuracy achieved is 88.51% on the test set. Figure 9 presents the loss and accuracy plots obtained for the training and validation sets.

#### 4.3.3 Analysis of the proposed architecture without quantum circuits

**Figure 10:**
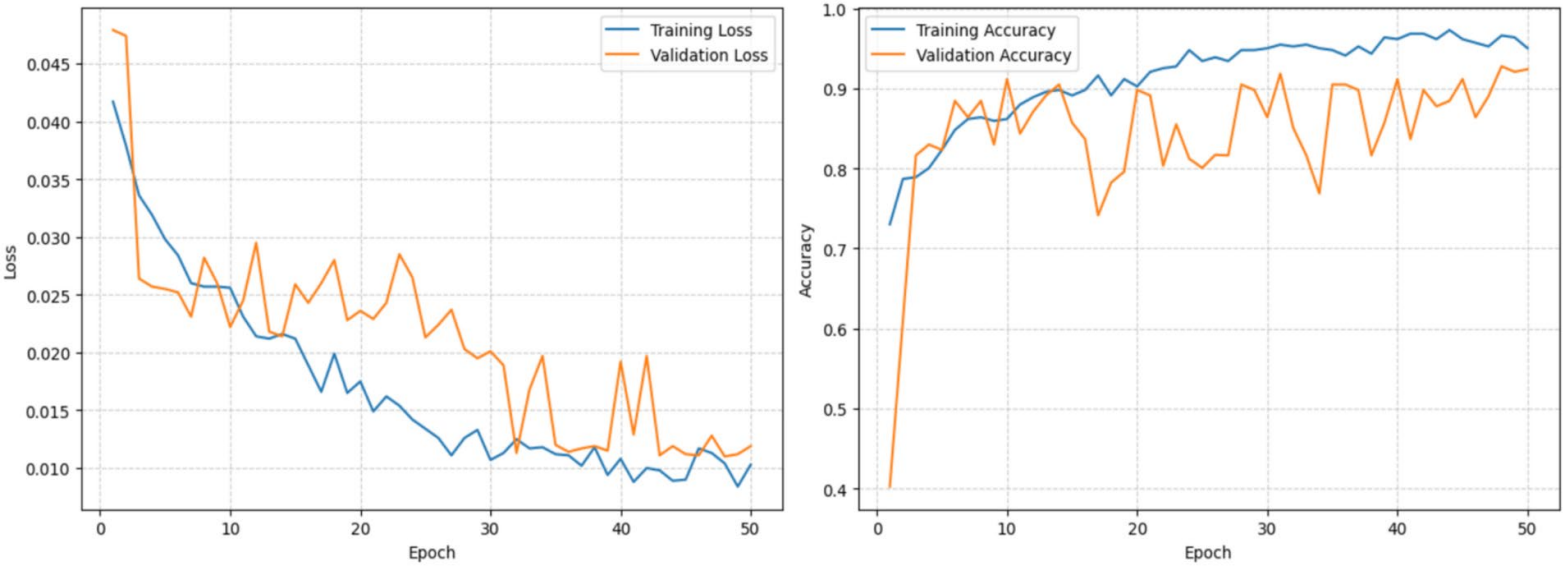
Loss and accuracy graphs of training and validation sets.

This experiment presents the performance obtained from using the proposed architecture by replacing all the quantum modules with equivalent classical neural components. The VQC in the TVSFE track was replaced with fully connected dense layers of equivalent output dimensionality. The VQFMN track was substituted with a classical multilayer perceptron employing ReLU activations to process the structured input features. This design preserves the overall dual-track multimodal architecture and fusion strategy while eliminating the quantum components. This isolates the contribution of the quantum modules to the overall classification performance. The network achieved an accuracy of 92.57% on the test set, with precision, recall, and F1-score of 93.27, 91.62, and 92.34%, respectively. Figure 10 presents the loss and accuracy plots from the training and validation sets.

#### 4.3.4 Analysis of the proposed architecture

**Figure 11:**
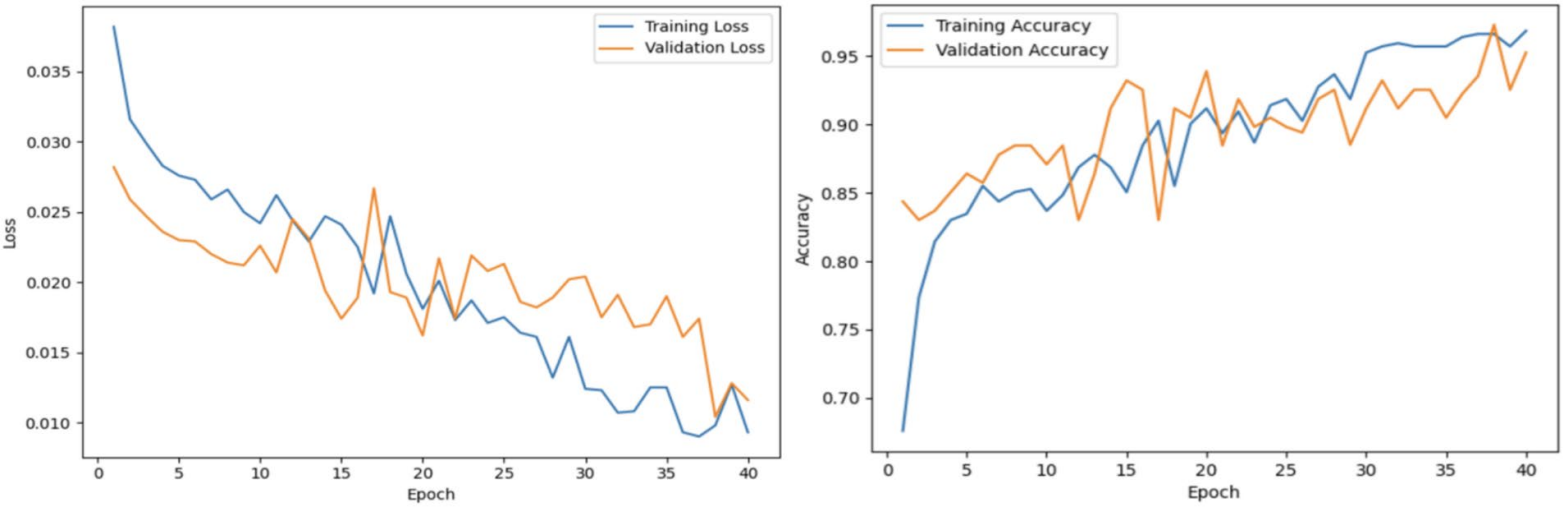
Analysis of proposed network using loss and accuracy graphs.

**Figure 12:**
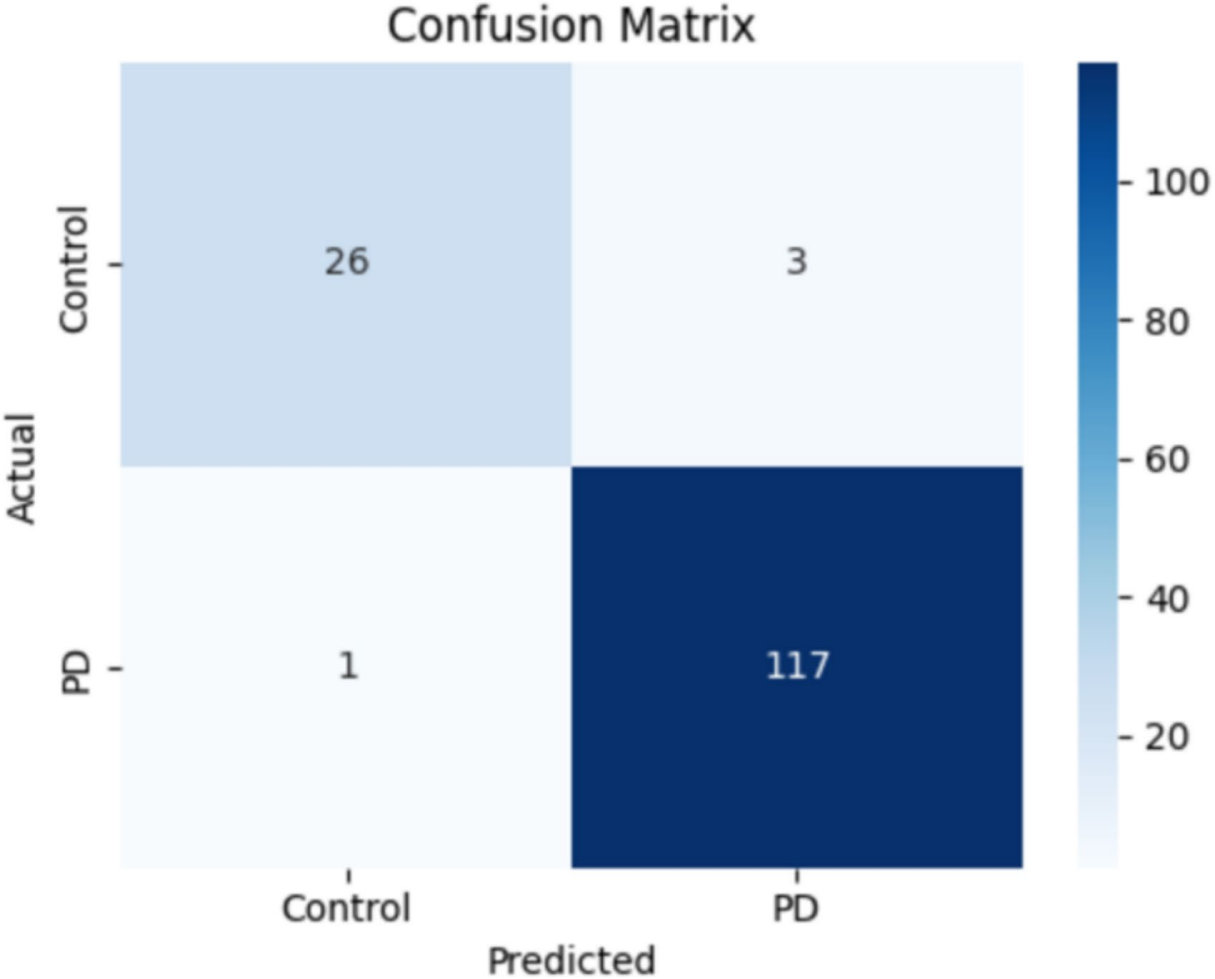
Confusion matrix depicting predictions of the proposed system.

This experiment demonstrates the performance of the overall proposed system. This overall system concatenates the outputs of TVSFE and VQFMN to produce combined multi-modal output results. The TVSFE utilizes GM and residual connections combined with CA and triplet attention mechanisms to extract spatially focused and contextual features. The VQFMN employed RY gates for encoding, followed by strongly entangled layers for capturing complex nonlinear patterns. The hybrid system achieved an accuracy of 97.28% on the test set. Figure 11 presents the performance graphs of the proposed system. Additionally, a confusion matrix was also generated to highlight the model’s precision and recall trade-offs across both classes. Figure 12 presents the confusion matrix of the results. Table 3 provides a summary of the ablation studies conducted.

**Table 3:** Summary of the ablation studies conducted in this study.

S. No	Experiment	Number of parameters	Accuracy (in %)	Precision (in %)	Recall (in %)	F1 Score (in %)	Inference Latency (in ms)	Memory (in MB)
1	TVSFE	1.1 M	93.93	94.27	93.92	94.04	33.94	5.90
2	VQFMN	402	88.51	86.97	87.84	86.70	19.00	$10^{-3}$
3	Proposed network without quantum circuits	1.19 M	92.57	93.27	91.62	92.34	15.34	3.59
4	Proposed Network	1.5 M	97.28	96.60	96.62	96.54	53.66	6.00

### 4.4 Cross-validation and statistical validation

A 5-fold cross-validation strategy was employed to evaluate the proposed system’s generalization capability. The complete dataset was partitioned into five mutually exclusive and approximately equal subsets. During each iteration, four folds were used for training, while the remaining folds were reserved for validation. This process was repeated five times to ensure that each subset serves as the validation set exactly once. This helps reduce the risk of overfitting and provides a reliable estimate of model performance of the model’s performance across different data partitions. The detailed cross-validation results are presented in Table 4. The model achieved consistent performance across all folds, demonstrating the stability of the proposed model and ensuring the results are not biased toward any subset of the dataset.

**Table 4:** Summary of 5-fold cross-validation.

Fold	Precision (in %)	Recall (in %)	F1-Score (in %)	Accuracy (in %)
1	95.92	95.06	95.50	95.95
2	96.57	96.95	96.70	96.96
3	95.94	96.29	96.07	96.64
4	95.89	95.80	95.81	96.40
5	95.27	97.83	96.27	96.96

**Figure 13:**
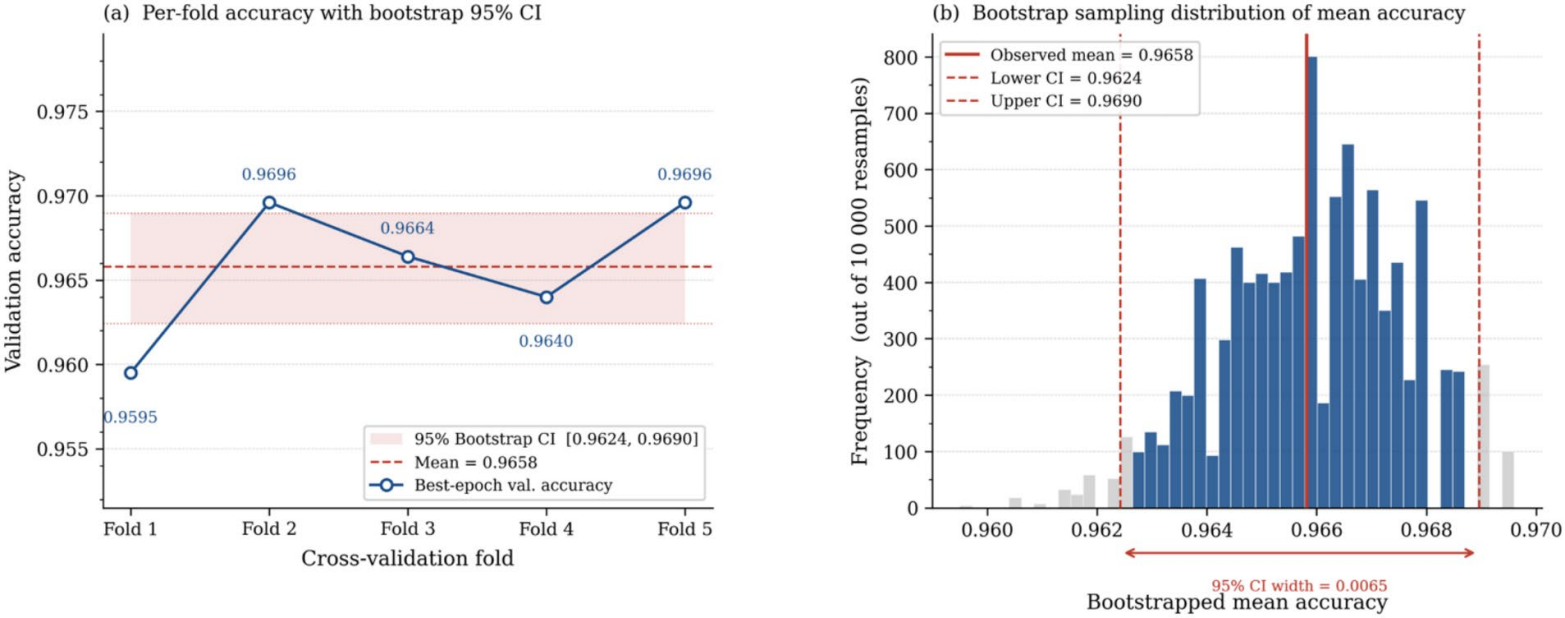
Statistical validation of model performance using 5-fold cross-validation and bootstrap analysis: **(a)** per-fold validation accuracy with 95% bootstrap confidence interval, and **(b)** bootstrap sampling distribution of mean accuracy across 10,000 resamples.

To further support the stability of the performance reported from the proposed network, a bootstrap confidence interval was employed. It was computed by pooling all per-sample predictions across the 5 folds and resampling with replacement 10,000 times to estimate the distributed mean accuracy. The analysis yielded a 95% bootstrap confidence interval of [0.9624, 0.9690], with an observed mean accuracy of 96.58% and width of 0.0065. The small interval and low variance across resample confirm that the performance of the proposed architecture is reproducible and stable. This confirms that the observed performance is unlikely to be due to incidental or marginal differences. The per-fold accuracy plot and the bootstrap sampling distribution of mean accuracy are presented in Figure 13.

## 5 Discussion

This section presents a detailed interpretation of the features learned by the proposed architecture and a comparison of the performance with existing studies and state-of-the-art networks.

### 5.1 Interpretation of the features

Feature interpretability methods were incorporated for both the image and structured data to understand the decision-making process of the proposed hybrid system. For the visual stream, Gradient-Weighted Class Activation Mapping (Grad-CAM) was applied to the output of the TVSFE. This helps to highlight the spatial regions that contributed most to the model’s predictions from the input images. It also reveals whether the network effectively focused on the key disease patterns from the input samples. The Grad-CAM visualizations are as shown in Table 5. The visualizations effectively focus on the irregular and distorted trajectories in both spiral and meander drawings of PD patients. The activated regions predominantly correspond to areas of stroke deviation, loop irregularity, and trace fragmentation, all of which are recognized clinical indicators of fine motor dysfunction in PD patients. The healthy individual’s heatmaps exhibited much smoother performance, with activations indicating a more evenly and uniformly applied pattern. This indicates that the TVSFE did successfully identify important markers of the disease, even when there were different forms of drawings and improved the reliability of diagnosis.

**Table 5:** Visualization of disease patterns using Grad-CAM.

S. No	Image class	Original sample	Grad-CAM visual
1	PD Patient (Spiral Drawing)	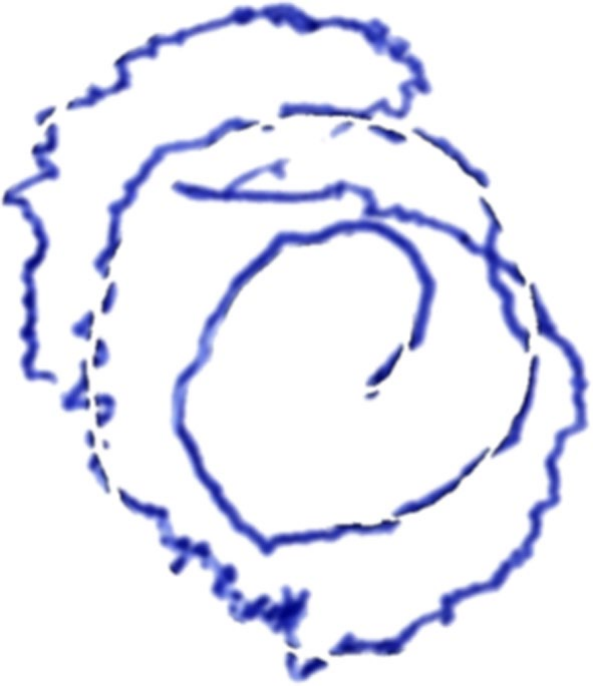	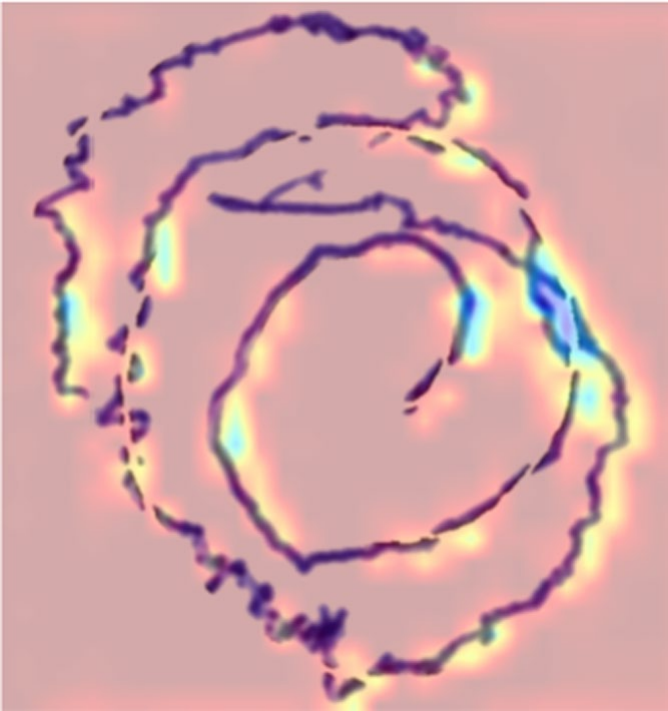
2	PD Patient (Meander Drawing)	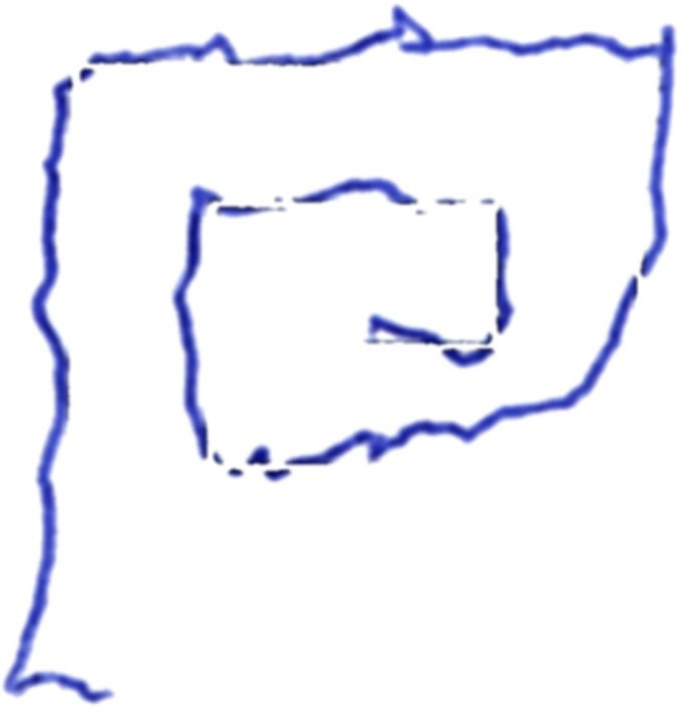	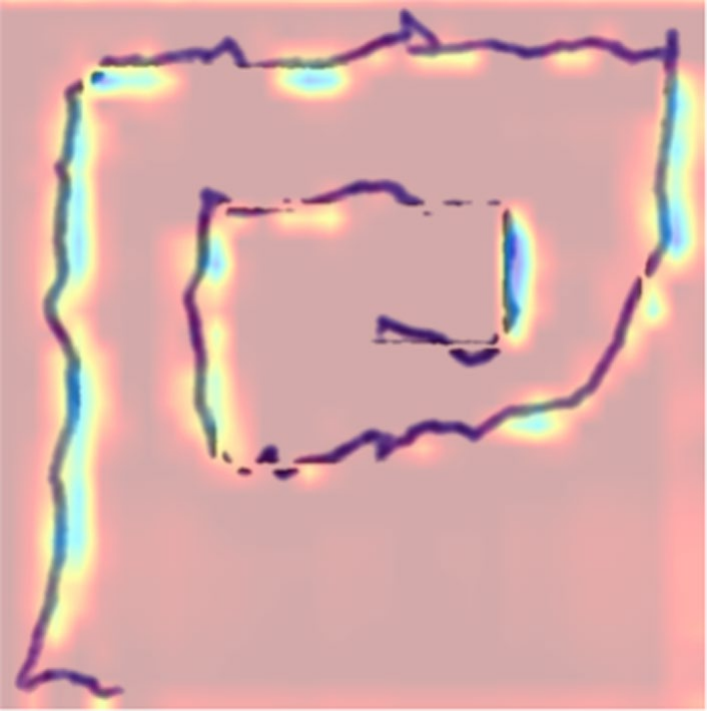
3	Healthy Subject (Spiral Drawing)	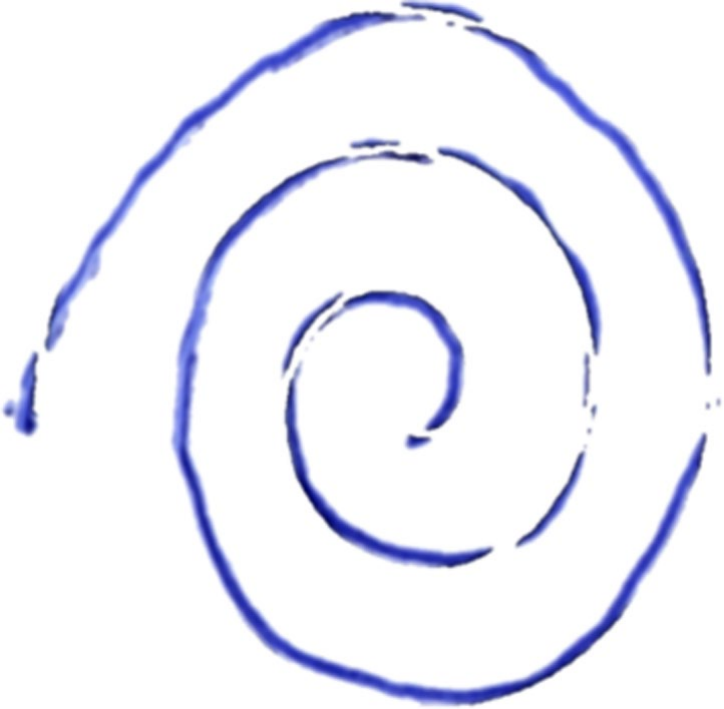	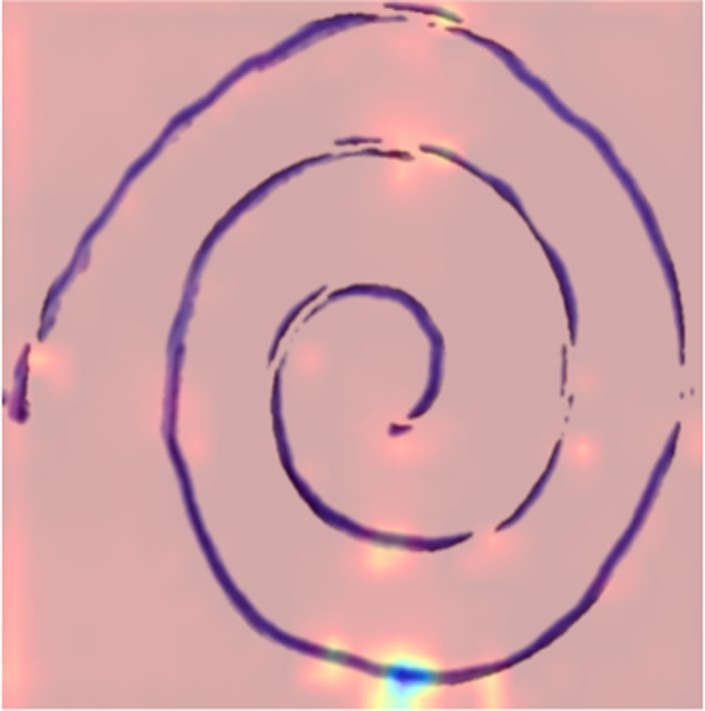
4	Healthy Subject (Meander Drawing)	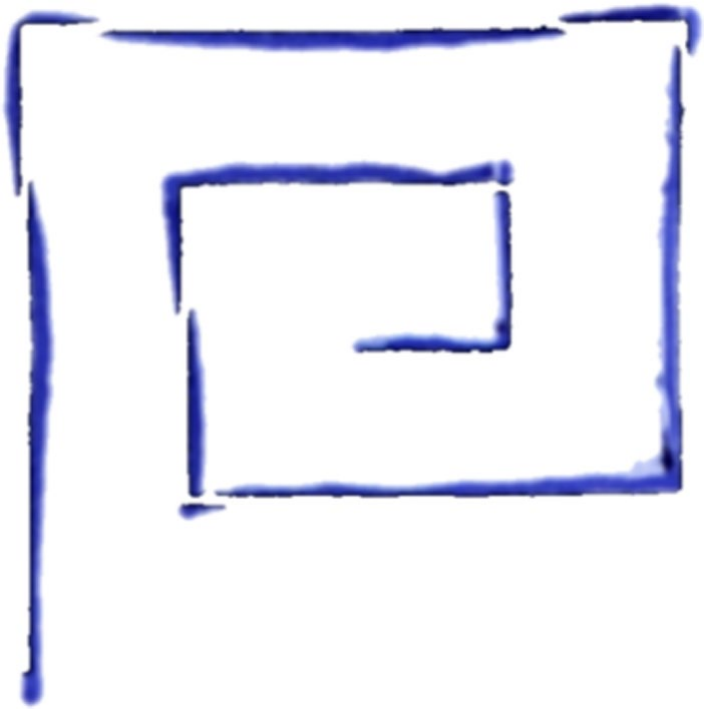	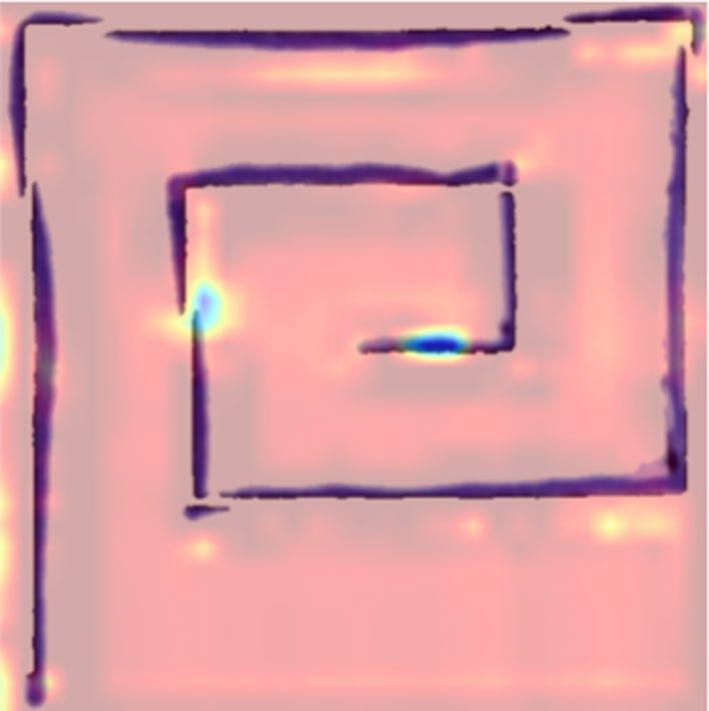

**Figure 14:**
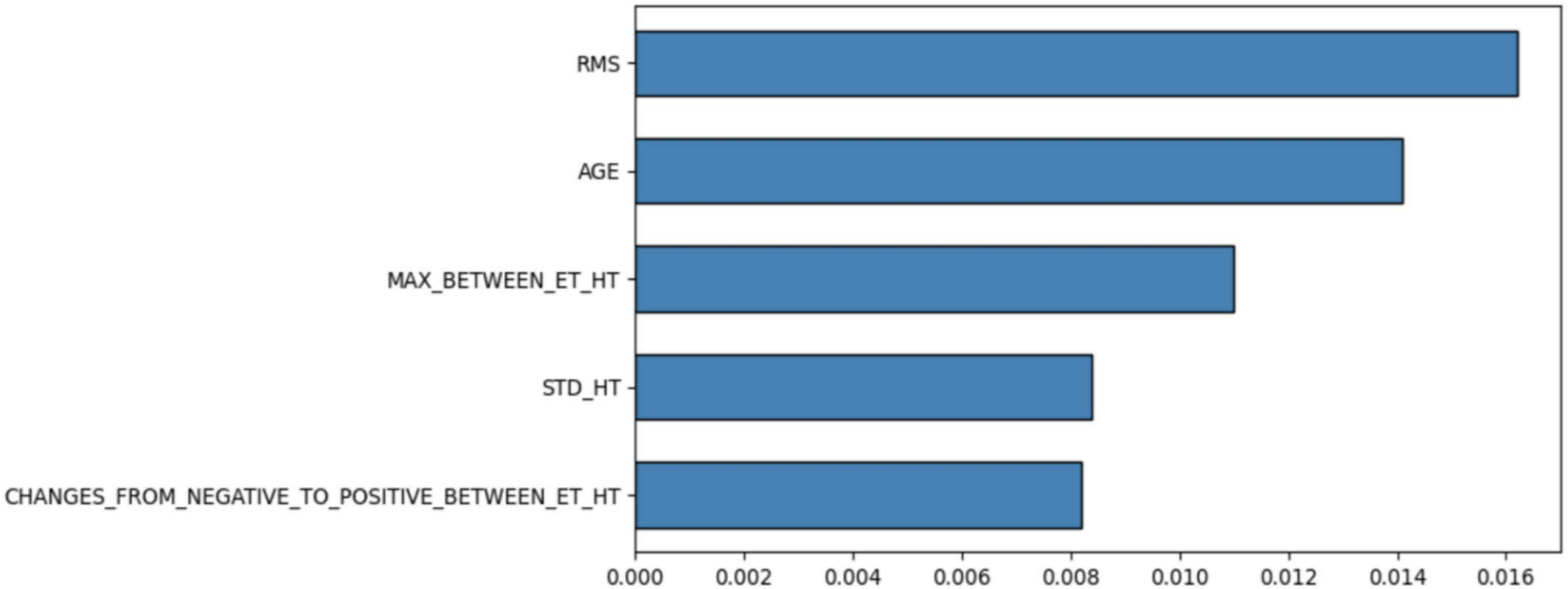
The top important features calculated based on perturbation-based sensitivity analysis using VQFMN architecture.

For the VQFMN track, a perturbation-based sensitivity analysis was performed to study the contributions that each feature made to the model’s predictions. The analysis provides the most distinct structured features that the quantum circuit used, while also providing insights into which parameters were most influential to the classification process. Analysis of feature importance level is shown in Figure 14 from the perturbation sensitivity analysis. The Root Mean Square (RMS) was identified to be the most important feature from the structured data. The RMS reflects the average deviation between the corresponding points on the handwritten trace and exam template. A higher RMS value indicates greater divergence between the patient’s trajectory and the template, potentially reflecting motor abnormalities or instability. Following RMS, age also was identified as the most important feature, aligning with its well-established role as a demographic risk factor in PD diagnosis.

### 5.2 Comparison with state-of-the-art models/networks

The performance analysis comparison of various CNN architectures with the proposed system is shown in Table 6. ViT has the highest parameter count of 86 million and achieves an accuracy of 87.84%. This indicates that model complexity does not always present efficient performance and results in longer inference per sample time. EfficientNet and ResNet were more parameter efficient with 91.89 and 92.57% accuracies, respectively. DenseNet produced the best accuracy of 94.59% among other state-of-the-art models. MobileNet and ShuffleNet had the fewest parameters, with 2.55 million and 1.52 million parameters respectively, while achieving good accuracy scores of 93.92 and 91.89%. The proposed system achieves 97.28% accuracy while maintaining the lowest parameter count. It also produced balanced performance across all evaluation metrics, while some models exhibit high recall and low precision scores, leading to more false positives in sensitive clinical applications.

**Table 6:** Comparison of the proposed system with other CNN and ML models.

Network/Model	Number of parameters (M)	Accuracy (in %)	Precision (in %)	Recall (in %)	F1-score (in %)	Inference time (in seconds)
ResNet	11.31	92.57	95.08	95.87	95.47	0.088
DenseNet	7.22	94.59	95.2	98.35	96.75	0.164
EfficientNet	4.34	91.89	98.23	91.74	94.87	0.053
MobileNet	2.55	93.92	96.67	95.87	96.27	0.035
ShuffleNet	1.52	91.89	96.58	93.39	94.96	0.019
ViT	86	87.84	90.55	95.04	92.74	0.631
Swin Transformer	27.58	88.51	87.77	88.51	87.82	0.005
GhostNet	3.9	83.11	81.57	83.11	82.09	0.003
Proposed system	1.5	97.28	96.60	96.62	96.54	0.781

### 5.3 Performance analysis with existing studies

Table 7 provides a comparison analysis of the proposed quantum system to existing studies that employed handwritten datasets. The results indicate that the proposed hybrid quantum network achieves an accuracy of 97.28%, surpassing previous studies. The combination of a quantum metaheuristics algorithm and a hybrid CNN yielded an accuracy of 96.70% and potential for quantum-inspired optimization to improve model performance. The study presented by Bahaddad et al. also integrated metaheuristics with DL, highlighting the benefits of hybrid algorithmic strategies. Transformers and custom CNNs have demonstrated similar accuracies ranging from 85 to 92%. This certainly highlights the success of developing a custom multimodal architecture to outline visual and structured features, resulting in a significant performance improvement.

**Table 7:** Comparative analysis of the proposed system with existing research.

S. No	Source	Method	Accuracy (in %)
1	Pereira et al. (2016a)	SVM, NB, OPF	76.44
2	Pereira et al. (2016b)	DL models	83.77
3	Zhu et al. (2022)	Continuous Convolution Network	85.7
4	Afonso et al. (2019)	Recurrence plot-based CNN	87
5	Wang et al. (2023)	CA-enhanced Swin Transformer	88.92
6	Parisi et al. (2021)	Hyper-sinh CNN	91
7	Bahaddad et al. (2022)	Metaheuristics with DL	93.3
8	Mansour (2024)	Quantum Mayfly Optimization + Hybrid CNN	96.70
9	Proposed system	Hybrid multimodal Quantum Network	97.28

### 5.4 Performance analysis with external datasets

The system proposed in the study was assessed on two external datasets to determine how well the model generalized. The results obtained highlight that the model adapts to unseen data, making it suitable across a range of clinical and research applications.

#### 5.4.1 Analysis of NewHandPD dataset

This section presents the performance analysis of the proposed system when evaluated on the NewHandPD dataset (Pereira et al., 2016b). The NewHandPD dataset is the updated version of the HandPD dataset and consists of 264 images collected from 66 patients. The dataset provides a balanced distribution of 160 and 104 samples from male and female participants, respectively. The proposed system was used to predict the data, to present its effectiveness in distinguishing PD and control subjects. The model achieved an accuracy of 95.45%, with precision, recall, and F1-scores of 95.46, 95.45, and 95.45%, respectively.

#### 5.4.2 Analysis of the spiral and wave drawing dataset

The proposed model was also tested on the dataset presented by Medasani (2023). The dataset consists of spiral and wave drawings collected from the participants. A total of 408 images were presented in the dataset with each class representing 204 images. The proposed TVSFE was evaluated to demonstrate its generalizability and accuracy in this dataset. The proposed network achieved an accuracy of 93.75%. Table 8 summarizes the overall performance of the proposed system across different datasets.

**Table 8:** Summary of performance analysis of the proposed network across different datasets.

Dataset	Accuracy (in %)	Precision (in %)	Recall (in %)	F1-Score (in %)
NewHandPD (Pereira et al., 2016b)	95.45	95.46	95.45	95.45
Spiral & Wave Dataset (Medasani, 2023)	94.75	93.75	93.75	93.75
HandPD (Pereira et al., 2016a)	97.28	96.60	96.62	96.54

### 5.5 Limitations and future works

This section describes the limitations of the proposed architecture. It also describes potential work to improve the performance of PD classification.

1. The quantum circuits used in the proposed architecture were simulated using classical hardware and therefore do not take into account the physical constraints of actual quantum systems. Existing quantum hardware is subject to limitations, such as qubit decoherence, limited connectivity of qubits, and gate noises that greatly impact the circuit performance. These all can result in reducing the proposed system performance and application accuracy and stability when used on an actual quantum computer.
2. The proposed architecture, while achieving strong classification performance, involves the integration of CNN-based attention mechanisms alongside quantum variational circuits, which introduces significant computational overhead. Additionally, the scalability of the system is limited by the increased use of computational resources for quantum circuits. As the qubit count increases, the simulations become computationally expensive, requiring memory-heavy and larger systems to train them. Real quantum hardware is needed to explore the true potential scalability, but such access currently remains scarce and error-prone.
3. The current study utilizes hand-drawn spiral and meander patterns which are accessible and non-invasive proxies for PD-related motor impairments. While these assessments have been adopted in literature, it is acknowledged that neuroimaging modalities such as DAT SPECT, DTI, and structural MRI can offer complementary insights by capturing direct changes associated with PD. Future studies could explore the integration of such imaging inputs as complementary tracks, improving the multimodal features which can potentially enrich the diagnostic representation.
4. The interpretability of the hybrid quantum system remains elusive, limiting clinical acceptance and trust in clinical settings. The lack of a standardized explainable quantum tool limits the complete understanding of the decisions taken by the model. Therefore, it is necessary for future research to develop interpretable frameworks for quantum systems that are important in high-stakes domains such as healthcare.

## 6 Conclusion

PD is one of the complex neurodegenerative diseases that progress gradually with subtle motor and non-motor symptoms. These systems often overlap with other conditions, making the conventional diagnostic approaches time-intensive and requiring well experienced experts to efficiently minimize human errors. This study addresses the limitations of the traditional methods and existing research by proposing a hybrid multimodal quantum-classical system that utilizes both visual and structured data to detect PD in patients efficiently. The proposed architecture incorporates two customized tracks, namely, the TVSFE and the VQFMN, to tackle image and structured modality, respectively. The TVSFE contains a hybrid structure with attention-based CNN mechanisms and a quantum circuit to process rich feature maps extracted from the diagnostic images. In parallel, the VQFMN processes the clinical demographic and the structured diagnostic input of the individuals using quantum entangled layers. In final stage, feature maps from both the tracks are fused to present a complementary and unified feature representation that is further processed to provide efficient classification results. The study also incorporated explainability frameworks such as Grad-CAM visualization and perturbation-based sensitivity analysis to interpret the decision-making of both the tracks. The network attained an accuracy of 97.28% in the testing set, outperforming other baselines and existing research. The proposed framework demonstrates potential for upcoming decision-support systems that can aid clinicians in neurological disorder analysis.

## Data Availability

Publicly available datasets were analyzed in this study. This data can be found at: https://wwwp.fc.unesp.br/~papa/pub/datasets/Handpd/.
